# Comparative cognition for conservationists

**DOI:** 10.1016/j.tree.2014.06.004

**Published:** 2014-09

**Authors:** Alison L. Greggor, Nicola S. Clayton, Ben Phalan, Alex Thornton

**Affiliations:** 1 Department of Psychology, University of Cambridge, Cambridge, UK; 2 Department of Zoology, University of Cambridge, Cambridge, UK; 3 Centre for Ecology and Conservation, University of Exeter, Penryn Campus, Exeter, UK

**Keywords:** cognition, animal conservation, perception, learning, aversive conditioning, imprinting

## Abstract

Every animal occupies a unique cognitive world based on its sensory capacities, and attentional and learning biases. Behaviour results from the interaction of this cognitive world with the environment. As humans alter environments, cognitive processes ranging from perceptual processes to learned behaviour govern animals’ reactions. By harnessing animals’ perceptual biases and applying insights from cognitive theory, we can purposefully alter cues to reduce maladaptive responses and shape behaviour. Despite the fundamental connection between cognition and behaviour, the breadth of cognitive theory is underutilised in conservation practice. Bridging these disciplines could augment existing conservation efforts targeting animal behaviour. We outline relevant principles of perception and learning, and develop a step-by-step process for applying aspects of cognition towards specific conservation issues.

## Highlights

• Animal behaviour affects conservation and is driven by underlying cognition.
• Using cognitive principles can modify behaviour across taxonomic groups.
• We discuss concepts previously unexplored in conservation contexts.
• We create a novel guide for applying cognition to diverse conservation issues.

## Why cognition?

**Figure 1:**
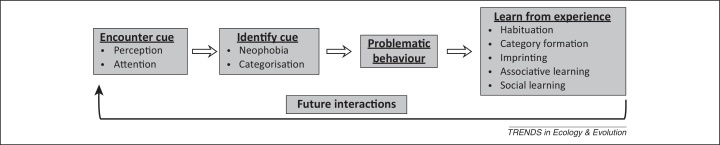
Cognition and the stages of problematic behaviour. The stages of interaction that an animal goes through to produce problematic behaviour are written in bold. The cognitive mechanisms that can be targeted at each stage are listed. Learning does not necessarily occur, but when it does it influences future interactions. Effective behavioural manipulations can involve intervention at various stages.

Ethology is an important component of conservation [1]. Behaviour drives ecological patterns, such as dispersal and predator–prey interactions, thereby affecting the distribution of species and influencing ecosystem functioning. Many urgent animal conservation issues (e.g., eradicating invasive species [2]) depend upon successfully manipulating behaviour. But what ultimately shapes behavioural patterns? Behaviour is an interaction with the environment stemming from what animals perceive, learn, remember, and decide to do; all of which make up cognition in its widest sense [3] (see Glossary). Cognitive mechanisms therefore underlie behavioural responses, and are central to understanding behaviour in conservation contexts (Figure 1).

Animal conservation incorporates diverse policies and wildlife management methods, and some, including reintroductions [4], trapping [5], invasive species mitigation [6], and deterrents [7] rely on manipulating animals’ behavioural responses. These interventions could be improved with insights from comparative cognition. For example, avian collisions with human-made structures kill millions of birds every year – including threatened and endangered species [8] – and are linked to population decline [9]. Existing solutions, like strategically placing [8], or altering structures [10], have had only limited success [11]. Crucially, wind farm deterrents will only be effective if they are reliably perceived, and rapidly learned; both of which are facets of cognition. Cognitive theory can thus help predict how best to manipulate and exploit attentional biases, innate responses, and learning tendencies to enhance conservation efforts. Because basic cognitive principles can be applied throughout the animal kingdom, these tactics can be used to address diverse problems.

**Figure 2:**
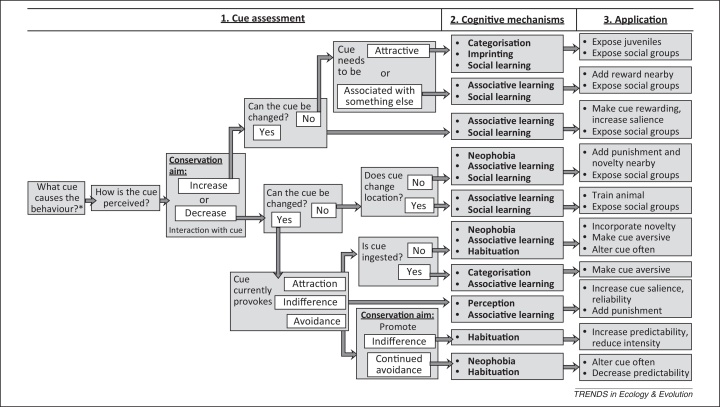
Applying cognition. Guides the reader through the three steps of cue manipulation to change a problematic behaviour: (1) cue assessment; (2) identifying relevant cognitive mechanisms; and (3) applying cognitive theory to the specific problem. See case studies and supplementary material for examples. When groups of stimuli occur, use the same cognitive strategy at every location and occurrence to promote generalisation.

Although elements of cognition have been explored in conservation contexts 12, 13, 14, discussions that integrate the breadth of cognitive theory in applied conservation contexts are lacking. Below, we outline the range of cognitive principles that can be used by conservationists, at each stage of problematic behaviour (Figure 1). Specifically, we discuss perceptual principles that influence behaviour towards novel cues, and emphasise the role of learning in determining repeated responses. Different mitigation tactics may be required for maladaptive behaviours that originate from attraction or aversion to novel cues. We conclude with a guide to applying these concepts (Figure 2) and with several case studies illustrating potential solutions.

## Cognition as adaptation

Animals possess perceptual biases and specialisations in learning and memory that have evolved in response to the specific challenges of their ancestral environments [3]. Human-induced rapid environmental change (HIREC [12]) generates evolutionarily novel cues and potentially imposes strong selection pressures on these biases and specialisations. Cognitive adaptations can therefore be as powerful as morphological adaptations in helping or hindering animals when environments change. For example, a cognitive mechanism that causes avoidance of novel food is as encumbering as a specialised feeding apparatus that prevents an animal from eating that food. Identifying the cognitive biases of target species requires stepping outside our own sensory experience and evaluating the saliency of novelty from the perspective of animals [15]. Even though the cognitive biases of all species are not perfectly catalogued, fundamental perceptual and learning theories are highly relevant across species.

## Perception of novelty

How animals perceive novel cues critically influences their response. Novel cues that resemble evolutionarily relevant cues are more likely to evoke common responses (i.e., the cue similarity hypothesis [12]) that can be adaptive (e.g., fleeing novel predators that resemble existing ones [16]). This helps explain why introduced species are more successful in novel environments that are similar to their ancestral ones [17]. However, when novel cues match relevant cues, but fail to produce beneficial outcomes, animals are at risk of perceptual errors and evolutionary traps (see [18] for a recent review)*.* For example, the colour, shape, and motion of plastic waste often resembles that of natural prey, provoking fishes, turtles, seabirds, and marine mammals to ingest them, with fatal consequences [19].

### Categorisation

Both adaptive and maladaptive responses to cue similarity can be explained through categorisation. Categorisation involves classifying or differentiating cues based upon perceptual or conceptual similarity [3], and allows novel cues to be processed and learned more quickly and efficiently [20]. Although some animals can categorise disparate cues, generally novel cues that perceptually overlap with known cues are more easily classified. For example, prey more easily categorise novel predators that resemble native ones [21]. However this same process can lead to damaging miscategorisation. For example, buprestid beetles (*Julodimorpha bakewelli*) are attracted to beer bottles whose colour and contours mimic those of their mates [22]. Miscategorisation could be prevented by designing bottles of different colours and textures (i.e., ‘cue disarming’ [18]).

Humans have long exploited perceptual and categorisation errors to shape behaviour. We take advantage of them in household pest control with bug zappers and poisonous baits, but we can also use them for conservation purposes. Insight into the aspects of cues that evoke inappropriate behaviour allows us to reduce perceptual errors [18]. For example, using lamps with larger wavelengths could help reduce the impact of human-made lights on moths [23], and simple alterations to lighthouses and oil rigs can prevent birds from succumbing to artificial light cues 24, 25. Nevertheless, conservationists need to explore solutions beyond reducing perceptual errors because they represent but a small fraction of possible cognitive manipulations. Fundamentally, much behaviour is not driven by automatic responses to cue similarity, but by experiences with cue novelty.

### Neophobia

Fearing or failing to fear human-made cues can generate problematic behaviour. Negative emotional responses to novel cues, termed neophobia, are adaptive in helping animals avoid unknown dangers [26]. However, when humans produce novelty, high levels of neophobia can prevent adaptive responses, such as inhibiting animals from incorporating new foods into their diet [27], whereas low neophobia levels can aid in invading novel habitats [28]. The extent to which neophobia produces avoidance behaviour depends upon the species [26], the individual's temperament [29], developmental stage, and experience 26, 30. Neophobia can be quantified in laboratory and field avoidance tests (e.g., [31]), therefore, measuring variation in neophobic behaviour within a population could predict how animals will respond to novel cues. With this information, the principles of neophobia can be applied to modify novel cues and increase or decrease fear responses.

Increasing fear responses can reduce human–animal conflict in farming and fishing contexts. Animals raid farms and steal catches, creating conflict with humans that results in needless culling and negative attitudes towards wildlife, often reducing support for local conservation programmes [32]. Capitalising on animals’ adaptive fear responses by amplifying biologically relevant surprise and danger signals can reliably deter animals from feeding [7], and tapping into neophobia could further enhance avoidance behaviour. For example, fear responses of animals to naturally aversive startle displays [33] and alarm calls [3] would be amplified if combined with cues that elicit neophobia, such as moving and changing objects [34]. Additionally, incorporating other naturally aversive stimuli into deterrents, such as noxious chemicals like chili powder [35] or quinine (e.g., [36]), might be effective (see [7]).

Although lessons from perception can manipulate initial reactions towards stimuli, shaping subsequent interactions requires an understanding of learning.

## Learning

Learning is a change in cognitive state that results from experience [3]. Learning is crucial to conservation because it can allow animals to acquire appropriate behavioural responses to novel cues [37] (Box 1). Basic learning abilities are ubiquitous, but what, when, and how animals learn depends upon several factors. Evolved learning biases can direct attention towards adaptive cues, but only if evolutionarily relevant cues are preserved [13]. Learning biases can favour certain sensory modalities. For example, animals more easily associate nausea with a taste than a shock or a light (Garcia effect [38]). Natural selection has directed attention towards taste cues around food because taste more reliably predicts the presence and quantity of toxins. Generally, experiences that are more biologically relevant and perceptually salient are learned faster than less relevant ones [3].

Box 1 **How learning can reduce impacts of invasive species**

Major goals of conservation biology are to preserve species and to maintain genetic diversity within species. Greater genetic diversity allows species to respond adaptively to future environmental changes, even if such adaptation is learning dependent [14]. However, novel selection pressures, such as those posed by HIREC, can reduce the genetic diversity of affected species.

Populations of many predatory species are at risk in Australia where the toxic cane toad (*Rhinella marina*) has invaded large portions of the country and is often mistaken as prey. Some predator populations, such as the red-bellied black snake (*Pseudechis porphyriacus*), have persisted through the selective survival of individuals that are morphologically preadapted with smaller jaws; restricting their ability to consume large enough toads to be poisoned [64]. Other species, such as the common planigale (*Planigale maculata*) (Figure I) and crimson spotted rainbow fish (*Melanotaenia duboulayi*) [36] use food aversion learning to avoid poisoning after ingesting nonfatal amounts of toad toxin [65]. Although the planigale and rainbow fish have still suffered losses – and comparisons between populations of rainbow fish in areas with and without invasive cane toads show evidence of selection for aversion learning [36] – their losses are less drastic than for toad-eating snakes [66], and less phenotypically discriminating than species that undergo rapid morphological evolution. Aversion learning requires initially ingesting a nonfatal amount of toxin, therefore, survival is determined more by the size of the toad or tadpole encountered than by a specific phenotype [67]. Therefore, learned behavioural responses could help maintain genetic diversity [67] (see Figure S3 in the supplementary material online).

**Figure I:**
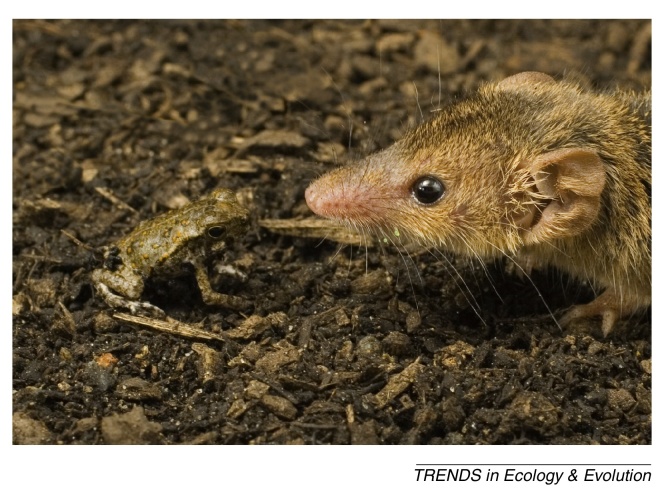
A planigale encountering a cane toad. Reproduced with permission from Jonathan Webb.

Although learning without human intervention might buffer certain species against diversity losses [67], actively encouraging learning could help species and/or individuals that might otherwise fail to make life-saving associations. This has become clear for another declining Australian predator, the northern quoll (*Dasyurus hallucatus*), that failed to learn about the toad not because they lack learning abilities, but because they hunt boldly, attacking large toads and suffering fatal first encounters. Reintroduction efforts have successfully trained quolls through aversive conditioning. Researchers coated small dead toads with nausea-inducing thiabendazole to foster an association of sickness with toads, thereby training released individuals to avoid natural encounters with the toads and survive [6]. It has been proposed that training baits could be aerially dropped in the wild to train populations about the toad before it arrives [6]. Through this method, targeting cognitive mechanisms to expedite learning could help protect a globally endangered species.

### Habituation

Habituation, measured as a decrease in response to a repeated cue, is considered the simplest form of learning, and allows animals to filter irrelevant information [39]. Habituation is often used to describe the process of behaviourally adapting to anthropomorphic disturbance across contexts ranging from chronic noise [40] to human visitors [41]. Different underlying processes can contribute to habituation between contexts, so animals might not tolerate shipping noise as readily as disruptions from tourists. The degree to which animals habituate has serious consequences for conservation programmes depending on the context. For example, crop deterrents will be less effective on animals that easily habituate, and animals that habituate poorly might be less tolerant to disturbances caused by habitat fragmentation.

Habituation relies on experiencing predictable cues [3] and can be prevented by amplifying differences in cues between presentations and timing presentations unpredictably. For example, randomly rotating crop deterrents between objects of different colours, sizes, and shapes, and by pairing them with different sounds will help prevent habituation (however, deterrents must also produce aversive experiences or cue variation will still fail to deter, e.g., [42]). In promoting habituation to minimise the effects of human disturbance, predictability should be maximised. For example, ecotourists in areas with disturbance-sensitive animals could be encouraged to wear similar clothes, follow similar paths, and only visit at specific times of day.

### Imprinting

Imprinting is a specialised form of learning that occurs during a short sensitive period in development to create strong preferences for one's own species [43], specific foods, habitats [44], or sites [43]. Imprinting can propagate parental behavioural patterns in future generations. For example, habitat imprinting can spread preferences for urban habitats, thereby facilitating animals’ urbanisation [45]. Imprinting on evolutionarily novel cues can cause maladaptive behaviours. For instance, zebra finches (*Taeniopygia guttata*) solicit an incorrect mate after imprinting upon a different species [46].

Imprinting manipulations can aid conservation efforts – such as translocation programmes that depend upon animals preferring suitable environments [47] – and are often used in salmonid (Salmonidae) release programmes [48]. Exposing animals to a particular stimulus during their sensitive phase, such as the postlarval period for many insects [44], can create life-long preferences. Additionally, imprinting can be used as tool to guide other desired behaviours. For example, the Whooping Crane Eastern Partnership successfully exploited filial imprinting to lead reintroduced whooping cranes (*Grus americana*) through their first migration. After being exposed to costumed people during early development, the birds imprinted on the costumes so faithfully that they followed an ultralight aircraft flown by their ‘foster mothers’ [49].

### Associative learning

Animals from nematodes to humans [50] can learn associations between cues to better predict and respond to events in their environment. Whether associations form depends on the timing between the behaviour and its consequence (contiguity), the reliability (contingency) and salience of the stimulus, and the biological appropriateness of the association [3]. The breadth and scope of associatively learned behaviour allows the following principles to be used in many contexts.

Associative learning occurs through classical or operant conditioning. In classical conditioning, an animal's natural reflex (unconditioned response; UR) toward a behavioural trigger (unconditioned stimulus; US) is associated with a novel cue (conditioned stimulus; CS), so that the novel cue elicits the response (i.e., creating a conditioned response; CR). Famously, Pavlov demonstrated that a dog will salivate (CR) at the sound of a bell (CS) if it reliably precedes food (US) [3], thereby learning to predict the occurrence of food.

Instead of creating associations between stimuli, operant conditioning creates associations between behaviour and its rewarding or unpleasant consequences. These associations increase or decrease the preceding behaviour, and can create novel behaviour as small variants in responses are positively or negatively reinforced. In conservation contexts, possible rewards and punishments inherent to the situation need be assessed, and unwanted rewards or punishments removed. Failing to evaluate cues can reinforce unwanted behaviour unintentionally. For instance, if predators gain access to fishing catches while a mildly irritating deterrent is broadcast, the deterrent will be associated with positive outcomes, making it a ‘dinner bell’ [51]. However, with careful planning, operant conditioning can be a highly effective conservation tool. For example, wildlife managers successfully reduced trappings of native species while managing feral cat populations through aversive conditioning by fostering associations between a negative cue (nausea-inducing chemicals in trapping baits) and the experience of feeding in the trap [5].

### Category learning

Categories based upon perceptual similarity can form by learning simple associations between common aspects of cues (cue generalisation) [52]. Miscategorisation of novel cues through cue generalisation can result in perceptual errors, which is why altering cues can directly change behaviour. If novel cues cannot be altered, miscategorisation can be prevented by changing the animal's categories through training using associative learning principles. For example, greater bilbies (*Macrotis lagotis*) were trained to categorise cats, an invasive species, as predators by associating a multimodal cat stimulus with an unpleasant handling experience and repeated predation attempts [53].

Some animals are capable of categorisation that does not hinge on perceptual similarity, but instead stems from associations between concepts, such as higher-order categorisation (perceptually dissimilar, e.g., grouping garbage bins and children in one broad ‘things that drop food’ category), and abstract categorisation (neither functionally nor perceptually similar, e.g., sameness versus differentness). Being able to classify novelty into biologically relevant categories might help some animals cope with the large number of unfamiliar cues in novel environments. For instance, learning safe versus unsafe categories could allow animals to minimise costly avoidance behaviours and use effective flight responses (e.g., selectively responding to specific unsafe humans as predators [54]).These complex forms of categorisation might facilitate efficient responses across diverse environments, but they require more presentations to learn than perceptually similar categories [55]. Therefore, limiting the amount of perceptual overlap between items prevents cue generalisation and forces animals to rely on conceptual categorisation; making learning about novelty more time consuming for some species and impossible for others. Preventing easy categorisation in this way can be desirable, for example, when designing traps for species monitoring. Altering the appearance, scent, and location of the trap will hinder animals from categorising them as dangerous, and allow more of them to be retrapped.

### Social learning

Social learning, the ability to learn from others, can spread novel behaviour faster than genetic change, and with fewer costs than individual learning [56]. Social learning can simply involve drawing attention towards a location or cue (i.e., local or stimulus enhancement), with subsequent positive reinforcement perpetuating future attention and behaviour towards that cue [3]. Therefore, attention toward small social cues can facilitate population-level behavioural changes [57].

As with all learning, social learning is constrained by animals’ cognitive biases. For example, monkeys will learn to fear snakes but not flowers when simultaneously presented with conspecific fear responses [58]. Social learning would be favoured over individual learning in situations where the latter might be dangerous or difficult [57]. Interacting with novel foods, predators, and environments is inherently risky; therefore, animals are liable to use social information when novelty arises. In conservation contexts, social learning can, for example, spread information about novel predators in reintroduction programmes [59], and increase the viability of reintroduced hatchery-reared fish [60]. Therefore, whenever possible, programmes should allow animals to see conspecifics or trainers performing behaviours they wish to encourage.

## Purposefully altering cues: a step-by-step process

Conservationists often lack sufficient knowledge of cognitive theory to implement successful behavioural manipulations. Ineffective interventions can result from overlooking the fact that different cognitive mechanisms influence behaviour during initial encounters with a cue than encounters after experience and learning (e.g., not accounting for rewarding experiences [51]). Although some conservation issues need only address the perceptual stage (e.g., reducing perceptual errors [23]), others require changes at multiple stages of the learning process. Understanding when and how to target specific cognitive processes involves an integrated approach to utilising cognitive mechanisms (Figure 1). Addressing a problematic behaviour involves three steps: (i) assessing the cue that triggers the behaviour from the perspective of the animal; (ii) identifying the cognitive processes relevant to the situation; and (iii) targeting those processes within the constraints of the context of the cue and the known cognitive biases of the animal. We have generated a flowchart to guide readers through these three steps (Figure 2). We outline several examples to demonstrate how these principles apply to actual conservation problems, with additional examples provided (see the supplementary material online). The guidelines are widely applicable beyond these cases.

### Context: mining noise disturbs wildlife

Animals flee the sound of mine blasts, wasting energy and feeding time, and fail to learn to ignore them. We seek to decrease the extent to which blasts cause avoidance behaviour, but the sounds of the blasts themselves cannot be altered (Step 1). Consequently, steps must be taken to promote habituation such that the blasts no longer cause alarm (Step 2). Detonating blasts at the same time daily can make cues more predictable and encourage habituation (Step 3).

### Context: reintroduced species fails to breed in ancestral habitat

The species is either not attending to cues in its ancestral habitat, or it is failing to categorise the habitat as suitable. We seek to increase interaction with cues in the habitat, but the habitat itself cannot be modified. Interventions must aim to increase the attractiveness of the habitat (Step 1). Imprinting on habitat cues will help animals categorise the habitat as suitable and preferences may be reinforced through social learning (Step 2). This may be achieved by exposing groups of animals to native habitat cues from an early age (e.g., [47]) (Step 3).

### Context: birds collide with wind turbines

The limited visual acuity of birds in anterior areas and attentional biases towards the ground often make them unaware of human-made structures when flying [10]. We aim to decrease interaction with turbines, but the intrinsic design of turbines cannot be altered (Step 1). Efforts should focus on creating ground-based deterrents that allow birds to learn to associate turbines with negative consequences without having to experience a collision. Learned avoidance may subsequently spread through flocks by social learning (Step 2). Pairing a visual signal with a surprising cue such as quick, unpredictable movement [33] or creating a multimodal cue, by adding noise [61], would focus the attention of the animal, while promoting avoidance behaviour and learning (Step 3).

## Concluding remarks

The unadulterated places left for wildlife are shrinking, imposing novel selection pressures on animals’ morphological and cognitive adaptations. Although cognition can seem daunting or irrelevant to those outside the field, we argue that it ultimately underlies much behaviour, and its exploitation in conservation contexts offers new ways to reduce human impacts. By focusing on key cognitive mechanisms, cues and experiences can be manipulated to improve the efficacy of behaviourally focused conservation efforts. These mechanisms are well-researched in the field of comparative cognition, yet rarely utilised in animal conservation.

Initiating dialogue between comparative cognition and conservation will allow for applications of cognitive theory to be further developed and tested. With shared conservation goals, comparative psychologists can direct their research towards species of conservation concern, and conservationists can benefit by applying new cognitive insights to difficult problems. Ultimately, the success of cognition-based efforts relative to other conservation strategies needs to be empirically tested, and the costs of implementing them considered [62]. Even if integrating cognitive theory only initially advances a few of the areas where it could be applied, the potential value of these collaborations should no longer be ignored.

**Glossary**

**Abstract categorisation**: sorting cues into groups whose components are neither functionally nor perceptually similar (e.g., a concept of sameness or differentness).
**Associative learning**: process through which an individual learns the relationship between two cues, or a cue and a behavioural response.
**Aversive conditioning**: form of operant conditioning that creates an association between a negative cue (such as fear or pain) and an unwanted behaviour.
**Categorisation**: process of classifying or differentiating cues based upon perceptual or conceptual similarity [3].
**Classical conditioning**: when an event (US) that normally triggers a reflex (UR) is associated with a cue (CS). If the cue comes to evoke the reflex (CR), the association has been learned. Also known as Pavlovian Conditioning [3].
**Cue similarity hypothesis**: animals will be more likely to respond to a novel cue the closer it mimics the cue their ancestors encountered [12].
**Evolutionary trap**: cue that appears more attractive to an individual despite being associated with lower fitness [63].
**Filial imprinting**: imprinting on the mother. Best studied in precocial birds.
**Garcia effect**: animals rapidly associate taste cues with illness, even when separated by hours, but do not learn to associate other cue types with illness. Highly robust to habituation. First described by John Garcia [38].
**Habituation**: decrease in response to a repeated cue that is independent of sensory fatigue.
**Higher-order categorisation**: sorting stimuli into groups that are not based upon perceptual similarity (e.g., placing cars and guns in a ‘danger’ category).
**HIREC**: human-induced rapid environmental change, defined by [12].
**Imprinting**: learned preference based on early experience during a sensitive phase that dictates behaviours involving parental recognition, and choices about food, mates, and habitat [43].
**Learning**: change in cognitive state that results from experience, and that can influence future behaviour [3].
**Local enhancement**: when the interaction of another individual with an object draws attention to that object.
**Neophobia**: fear of novelty.
**Operant conditioning**: often known as instrumental conditioning; increasing or decreasing a behaviour because it is associated with a reward or punishment.
**Perceptual error**: interpreting a cue incorrectly: in the wrong context or through misidentification.
**Social learning**: learning from the behaviour or products of others.
**Stimulus or Cue enhancement**: when the interaction of another individual with an object draws attention to that object.

## References

[1] Candolin U., Wong B.B.M. (2012). Behavioural Responses to a Changing World; mechanisms and consequences.

[2] Sutherland W.J. (2014). A horizon scan of global conservation issues for 2014. Trends Ecol. Evol..

[3] Shettleworth S. (2010).

[4] Urbanek R.P. (2005). Reintroduction techniques: post-release performance of 54 sandhill cranes (1) released into wild flocks and (2) led on migration by ultralight aircraft. Proc. North Am. Crane Work.

[5] Phillips R.B., Winchell C.S. (2011). Reducing nontarget recaptures of an endangered predator using conditioned aversion and reward removal. J. Appl. Ecol..

[6] O’Donnell S. (2010). Conditioned taste aversion enhances the survival of an endangered predator imperilled by a toxic invader. J. Appl. Ecol..

[7] Schakner Z.A., Blumstein D.T. (2013). Behavioral biology of marine mammal deterrents: A review and prospectus. Biol. Conserv..

[8] Drewitt A.L., Langston R.H.W. (2008). Collision effects of wind-power generators and other obstacles on birds. Ann. N. Y. Acad. Sci..

[9] Phipps W.L. (2013). Do power lines and protected areas present a catch-22 situation for cape vultures (*Gyps coprotheres*)?. PLoS ONE.

[10] Alonso J.C. (1994). Mitigation of bird collisions with transmission lines through groundwire marking. Biol. Conserv..

[11] Martin G.R. (2011). Understanding bird collisions with man-made objects: a sensory ecology approach. Ibis (Lond. 1859).

[12] Sih A. (2011). Evolution and behavioural responses to human-induced rapid environmental change. Evol. Appl..

[13] Brown C., Candolin U., Wong B. (2012). Behavioural Responses to a Changing World; Mechanisms and Consequences.

[14] Sih A. (2013). Understanding variation in behavioural responses to human-induced rapid environmental change: a conceptual overview. Anim. Behav..

[15] Van Dyck H. (2012). Changing organisms in rapidly changing anthropogenic landscapes: the significance of the “Umwelt”-concept and functional habitat for animal conservation. Evol. Appl..

[16] Blumstein D.T. (2006). The multipredator hypothesis and the evolutionary persistence of antipredator behavior. Ethology.

[17] Blackburn T.M., Duncan R.P. (2001). Determinants of establishment success in introduced birds. Nature.

[18] Robertson B.a. (2013). Ecological novelty and the emergence of evolutionary traps. Trends Ecol. Evol..

[19] Derraik J.G.B. (2002). The pollution of the marine environment by plastic debris: a review. Mar. Pollut. Bull..

[20] Wasserman E.A. (1995). The conceptual abilities of pigeons. Am. Sci..

[21] Ferrari M.C.O. (2007). Generalization of learned predator recognition: an experimental test and framework for future studies. Proc. Biol. Sci..

[22] Gwynne D.T., Rentz D.C.F. (1983). Beetles on the bottle: male buprestids mistake stubbies for females (*Coleoptera*). J. Aust. Entomol. Soc..

[23] Van Langevelde F. (2011). Effect of spectral composition of artificial light on the attraction of moths. Biol. Conserv..

[24] Poot H. (2008). Green light for nocturnally migrating birds. Ecol. Soc..

[25] Jones J., Francis C.M. (2003). The effects of light characteristics on avian mortality at lighthouses. J. Avian Biol..

[26] Greenberg R., Mettke-Hofmann C., Nolan V., Thompson C.F. (2001).

[27] Marples N.M. (1998). Responses of wild birds to novel prey: evidence of dietary conservatism. Oikos.

[28] Sol D. (2011). Exploring or avoiding novel food resources? The novelty conflict in an invasive bird. PLoS ONE.

[29] Réale D. (2007). Integrating animal temperament within ecology and evolution. Biol. Rev. Camb. Philos. Soc..

[30] Marples N.M. (2007). Deactivation of dietary wariness through experience of novel food. Behav. Ecol..

[31] Seferta A. (2001). Learning differences between feral pigeons and zenaida doves: the role of neophobia and human proximity. Ecology.

[32] Hill C.M. (2002). Human-Wildlife Conflict: Identifying the Problem and Possible Solutions. (Albertine Rift Technical Report Series, Vol. 1).

[33] Olofsson M. (2012). Deimatic display in the European swallowtail butterfly as a secondary defence against attacks from great tits. PLoS ONE.

[34] Corey D.T. (1978). The determinants of exploration and neophobia. Neurosci. Biobehav. Rev..

[35] Sitati N.W., Walpole M.J. (2006). Assessing farm-based measures for mitigating human-elephant conflict in Transmara District, Kenya. Oryx.

[36] Caller G., Brown C. (2013). Evolutionary responses to invasion: cane toad sympatric fish show enhanced avoidance learning. PLoS ONE.

[37] Garcia T.S. (2012). Antipredator behavior of american bullfrogs (*Lithobates catesbeianus*) in a novel environment. Ethology.

[38] Garcia J. (1974). Behavioral regulation of the milieu interne in man and rat. Science.

[39] Rankin C.H. (2009). Habituation revisited: an updated and revised description of the behavioral characteristics of habituation. Neurobiol. Learn. Mem..

[40] Anderson P.A. (2011). Sound, stress, and seahorses: the consequences of a noisy environment to animal health. Aquaculture.

[41] Ellenberg U. (2009). Habituation potential of yellow-eyed penguins depends on sex, character and previous experience with humans. Anim. Behav..

[42] Muirhead S. (2006). Roo-Guard^®^ sound emitters are not effective at deterring tammar wallabies (*Macropus eugenii*) from a source of food. Wildl. Res..

[43] Immelmann K. (1975). Ecological significance of imprinting and early learning. Annu. Rev. Ecol. Syst..

[44] Davis J.M. (2008). Patterns of variation in the influence of natal experience on habitat choice. Q. Rev. Biol..

[45] Evans K.L. (2010). A conceptual framework for the colonisation of urban areas: the blackbird *Turdus merula* as a case study. Biol. Rev. Camb. Philos. Soc..

[46] Bischof H-J., Clayton N. (1991). Stabilization of sexual preferences by sexual experience in male zebra finches taeniopygia guttata castanotis. Behaviour.

[47] Binder D. (2013). Emergence, growth, ageing and provisioning of Providence petrel (*Pterodroma solandri*) chicks: implications for translocation. Emu.

[48] Brown C., Day R.L. (2002). The future of stock enhancements: lessons for hatchery practice from conservation biology. Fish Fish..

[49] Urbanek R.P. (2010). Winter release and management of reintroduced migratory Whooping Cranes Grus americana. Bird Conserv. Int..

[50] Heyes C. (2012). Simple minds: a qualified defence of associative learning. Philos. Trans. R. Soc. Lond. B: Biol. Sci..

[51] Carretta J.V., Barlow J. (2011). Long-term effectiveness, failure rates, and “dinner bell” properties of acoustic pinners in a gillnet fishery. Mar. Technol. Soc. J..

[52] Soto F.A., Wasserman E.A. (2010). Error-driven learning in visual categorization and object recognition: a common-elements model. Psychol. Rev..

[53] Moseby K.E. (2012). Can predator avoidance training improve reintroduction outcomes for the greater bilby in arid Australia?. Anim. Behav..

[54] Levey D.J. (2009). Urban mockingbirds quickly learn to identify individual humans. Proc. Natl. Acad. Sci. U.S.A..

[55] Katz J.S. (2007). Issues in the comparative cognition of abstract-concept learning. Comp. Cogn. Behav. Rev..

[56] Boyd R., Richerson P. (1985).

[57] Thornton A., Clutton-Brock T. (2011). Social learning and the development of individual and group behaviour in mammal societies. Philos. Trans. R. Soc. Lond. B: Biol. Sci..

[58] Cook M., Mineka S. (1989). Observational conditioning of fear to fear-relevant versus fear-irrelevant stimuli in rhesus monkeys. J. Abnorm. Psychol..

[59] Griffin A.S. (2004). Social learning about predators: a review and prospectus. Learn. Behav..

[60] Brown C., Laland K.N. (2001). Social learning and life skills training for hatchery reared fish. J. Fish Biol..

[61] Partan S.R. (2009). Wild tree squirrels respond with multisensory enhancement to conspecific robot alarm behaviour. Anim. Behav..

[62] Sutherland W.J. (2004). The need for evidence-based conservation. Trends Ecol. Evol..

[63] Schlaepfer M.A. (2002). Ecological and evolutionary traps. Trends Ecol. Evol..

[64] Phillips B., Shine R. (2006). An invasive species induces rapid adaptive change in a native predator: cain toads and black snakes in Australia. Proc. R. Soc. B: Biol. Sci..

[65] Webb J. (2008). A native dasyurid predator (common planigale, *Planigale maculata*) rapidly learns to avoid a toxic invader. Aust. Ecol..

[66] Covacevich J., Archer M. (1975). The distribution of the cane toad, *Bufo marinus*, in Australia and its effect on indigenous vertebrates. Mem. Queensl. Museum.

[67] Brown R.L. (2013). Learning, evolvability and exploratory behaviour: extending the evolutionary reach of learning. Biol. Philos..

