# Supplementary material for: Comparative cognition for conservationists
Source: Trends Ecol Evol. 2014 Sep;29(9):489–95. doi: 10.1016/j.tree.2014.06.004 (PMC4153814; doi:10.1016/j.tree.2014.06.004)
Supplement: Supplementary file 1 [file mmc1.doc]

**Supplementary Material** 
**Comparative Cognition for Conservationists**

Alison L. Greggor^1^, Nicola S. Clayton^1^, Ben Phalan^2^, and Alex Thornton^3^

^1^Department of Psychology, University of Cambridge

^2^Department of Zoology, University of Cambridge

^3^Centre for Ecology and Conservation, University of Exeter, Penryn Campus

Corresponding authors: Greggor, A.L. ([alg61@cam.ac.uk](mailto:alg61@cam.ac.uk)), Thornton, A. ([alex.thornton@exeter.ac.uk](mailto:alex.thornton@exeter.ac.uk))

**Extended examples illustrating how to apply cognition**

*Context: Vessel strikes endanger whales* *(Figure S1)*

Whales are fatally struck by ships because underwater engine noise is difficult to locate, and vessels move too quickly to avoid [S1] (Step 1). Two separate behavioural manipulations can be attempted: one targeting ships as the focal cue, the other targeting the prescribed shipping lanes in which they travel. If ships can be fitted with a directional short range auditory or pinger deterrent (e.g. [S2]) (Step 2) they may be easier to perceive and avoid, promoting associations between the deterrent and approaching ships (Step 3) (Figure S1A). If these same deterrents are subsequently attached to buoys along prescribed, unchanging shipping lanes (Step 2), whales should learn to avoid those areas (Step 3) (Figure S1B).

**Figure S1. Reducing vessel strikes**

**
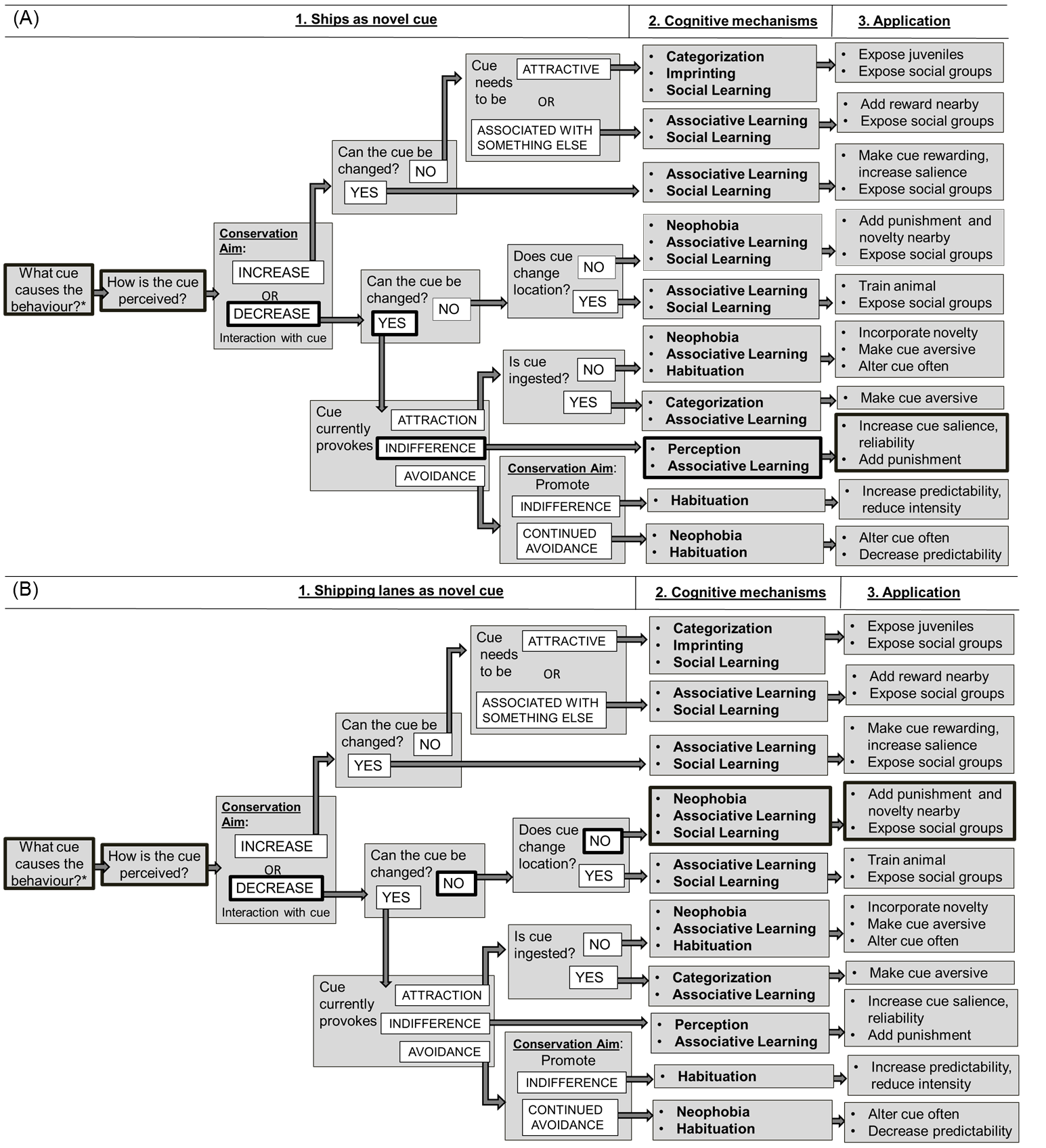
**

**Figure S1:** Strategies can be applied to multiple cues to change a single behaviour

*Context: Animals fail to use wildlife corridor over road (Figure S2)*

Animals avoid wildlife corridors because they seem irrelevant or dangerous (Step 1). To maximize the use of corridors (Step 2), design them to use biologically relevant safety signals such as vegetation cover to increase the attractiveness of the passage. Offering positive reinforcement such as food will help, as will allowing animals to see others cross safely (Step 3).

**Figure S2: Increasing use of wildlife corridor**

**
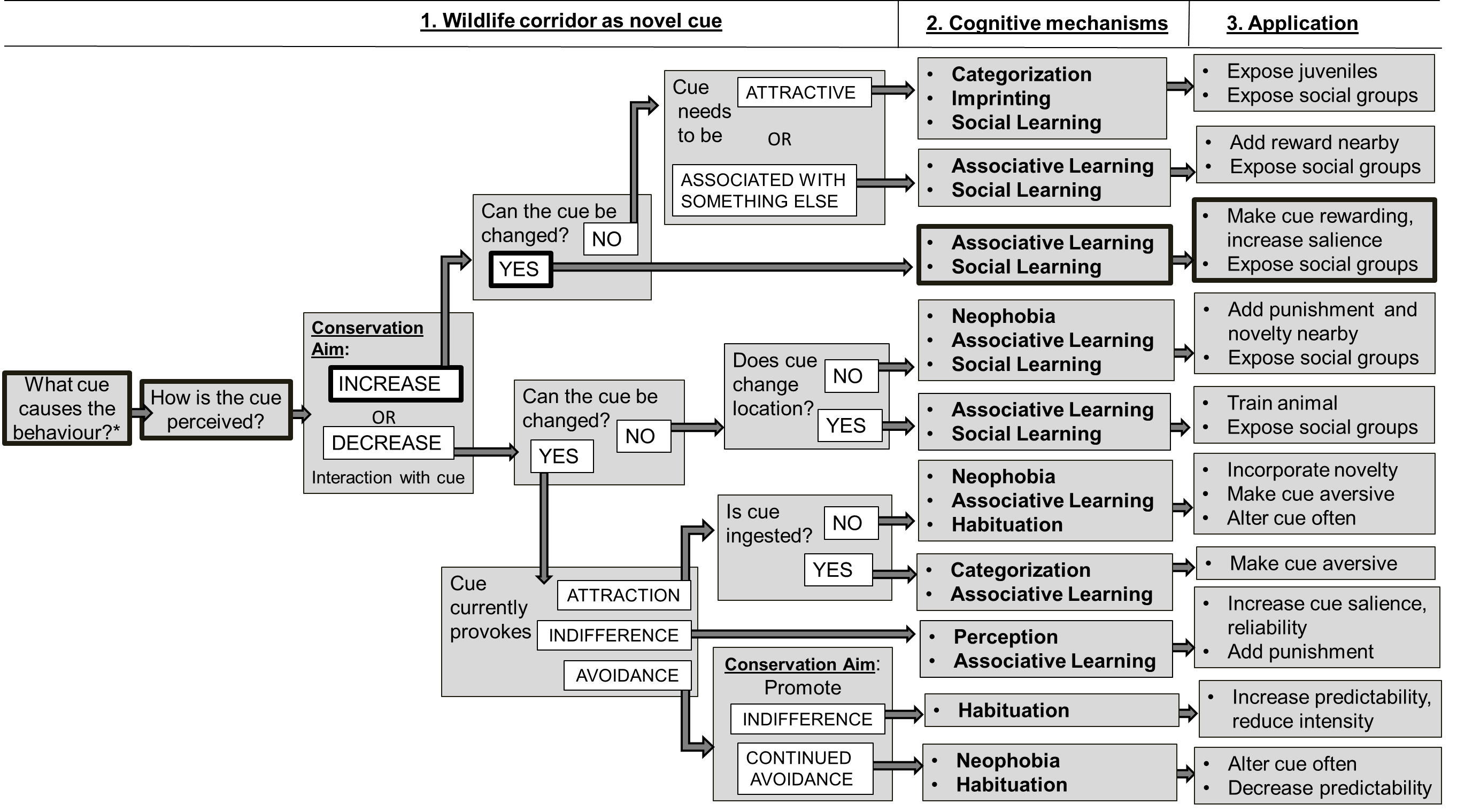
**

*Context: Toxic invasive species decimates local predator populations (Figure S3)*

Predators need to stop treating the invader as prey, because the invader cannot be removed (Step 1). Use aversive conditioning (Step 2) with non-lethal amounts of toxin as a negative cue, associated with the invader (i.e. deploy poisoned decoy baits [S3]). In species that can socially learn food aversions (e.g. [S4]), allow others to witness sickness or avoidance (Step 3).

**Figure S3: Reducing poisoning by invasive species**

**
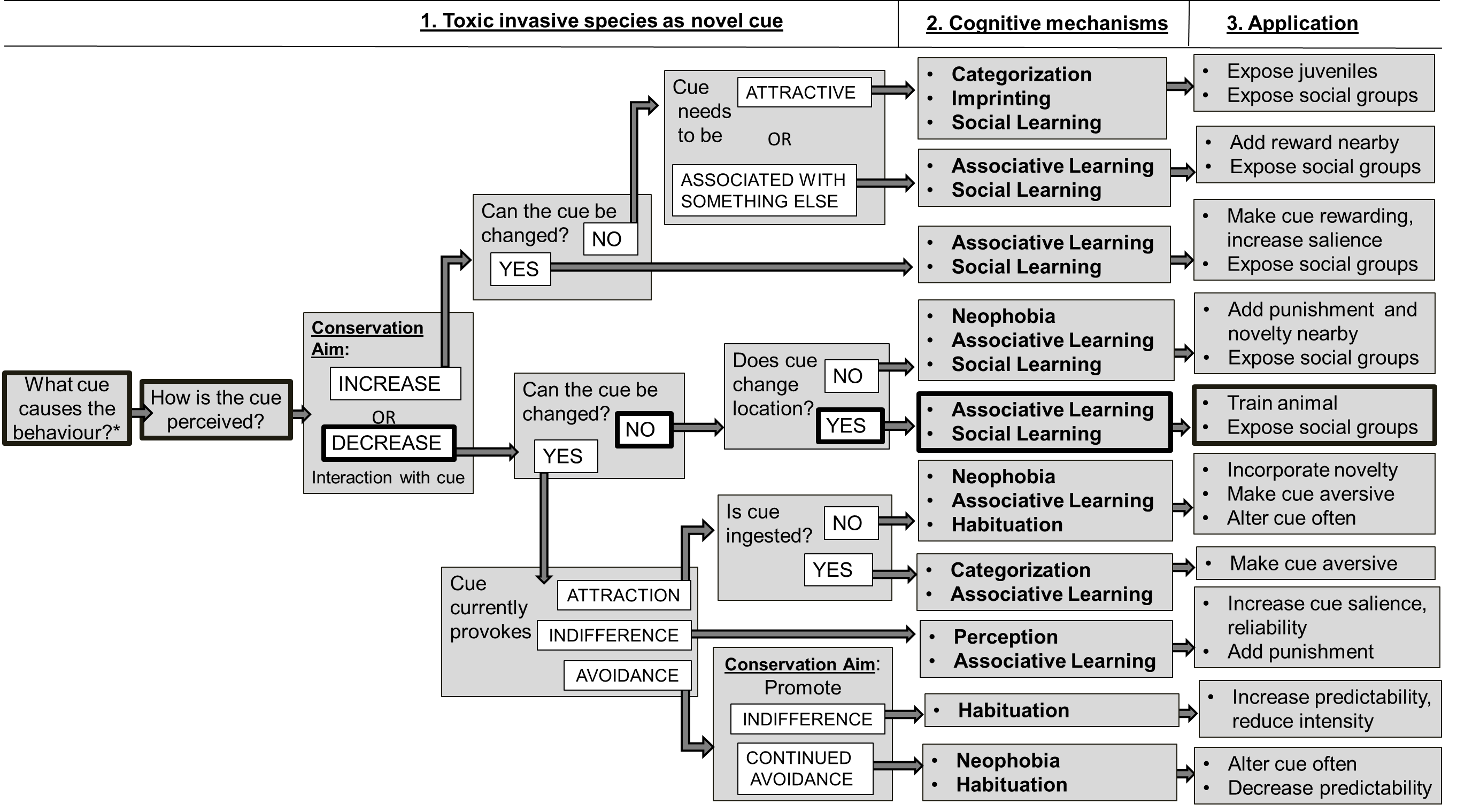
**

*Context: Invasive species removal program catches native species (Figure S4)*

Native animals enter the traps, wasting time and resources (Figure S4A; Step 1). Recaptures of unwanted species can be prevented by making interactions with the traps an aversive experience (Step2). Incorporating an aversive taste into the bait [S5], and broadcasting alarm calls of the native species would both create negative experiences. Each of the cues created by these tactics can then be assessed separately to ensure they will be effective over multiple encounters. Since taste aversions are not susceptible to habituation, they do not need changing (Figure S4B). Since alarm calls are, different sets of alarm calls should be used and the timing of their presentation should be varied (Figure S4C).

**Figure S4: Reducing native by-catch
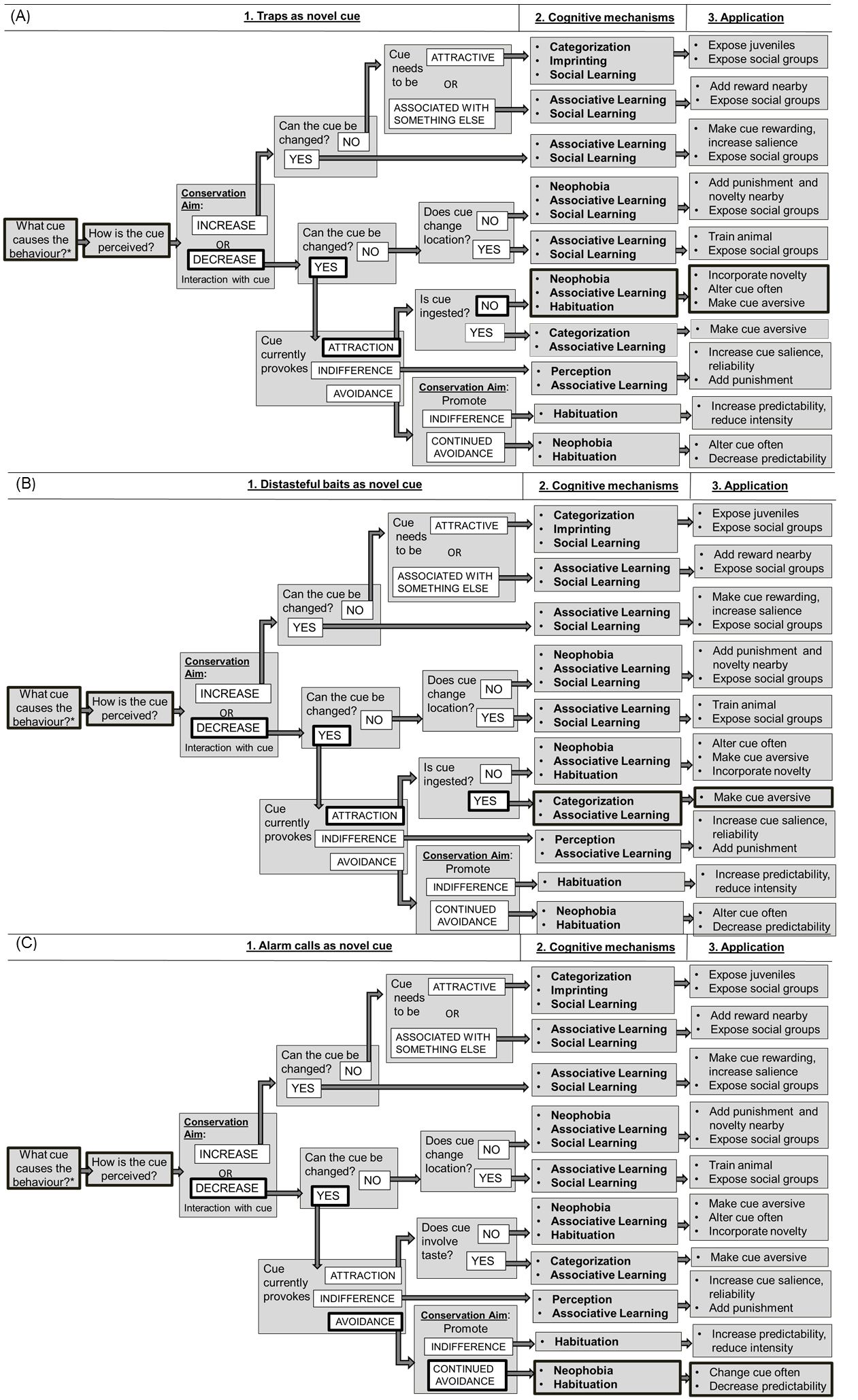
**

Figure S4: Different types of cues with can be employed at the same location

**References**

S1 Laist, D.W. *et al.* (2001) Collisions between ships and whales. *Mar. Mammal Sci.* 17, 35–75

S2 Schakner, Z.A. and Blumstein, D.T. (2013) Behavioral biology of marine mammal deterrents: A review and prospectus. *Biol. Conserv.* 167, 380–389

S3 O’Donnell, S. *et al.* (2010) Conditioned taste aversion enhances the survival of an endangered predator imperilled by a toxic invader. *J. Appl. Ecol.* 47, 558–565

S4 Johnston, A.N.B. *et al.* (1998) Observation learning in day-old chicks using a trial passive avoidance learning paradigm. *Anim. Behav.* 56, 1347–1353

S5 Phillips, R.B. and Winchell, C.S. (2011) Reducing nontarget recaptures of an endangered predator using conditioned aversion and reward removal. *J. Appl. Ecol.* 48, 1501–1507
